# Supplementary material for: Vaginal microbiome Lactobacillus crispatus is heritable among European American women
Source: Commun Biol. 2021 Aug 5;4:872. doi: 10.1038/s42003-021-02394-6 (PMC8342574; doi:10.1038/s42003-021-02394-6)
Supplement: Supplementary file 2 — Supplementary Information [file 42003_2021_2394_MOESM2_ESM.pdf]

Supplementary Information

Supplemental Table 1. Heritability estimates for predominant taxa by self-reported ancestry in women below the average age of menopause onset (< 51 years old).

Taxa	Ancestry	Component	Standardized Estimate	Unstandardized Estimate	95% LI	95% UI
Lactobacillus crispatus	African	A	0.2707	0.0794	-0.0635	0.2483
		E	0.7293	0.2141	0.1161	0.4130
	European	A	0.3731	0.1602	0.0596	0.2813
		E	0.6269	0.2691	0.1943	0.3819
Lactobacillus iners	African	A	0.1527	0.0454	-0.0993	0.2108
		E	0.8473	0.2516	0.1393	0.4697
	European	A	0.0798	0.0205	-0.0385	0.0824
		E	0.9202	0.2369	0.1762	0.3258
Gardnerella vaginalis	African	A	0.1073	0.0094	-0.0323	0.0550
		E	0.8927	0.0780	0.0445	0.1428
	European	A	-0.1730	-0.0169	-0.0432	0.0076
		E	1.1730	0.1143	0.0842	0.1580

LI = Lower Interval; UI = Upper Interval; A = additive genetic component; E = unique environmental component

Supplemental Table 2a. Model fit statistics for *L. crispatus* in the European American sample.

Model	Comparison	Parameters	-2*Log Likelihood	DF	AIC	Log Likelihood Difference	DF Difference	P-value
ACE	-	4	461.544	240	-18.456	NA	NA	NA
AE	ACE	3	463.562	241	-18.438	2.018	1	0.155
CE	ACE	3	467.132	241	-14.868	5.588	1	0.018
E	ACE	2	474.536	242	-9.464	12.992	2	0.002

Supplemental Table 2b. Model fit statistics for *L. iners* in the European American sample.

Model	Comparison	Parameters	-2*Log Likelihood	DF	AIC	Log Likelihood Difference	DF Difference	P-value
ACE	-	4	353.389	240	-126.611	NA	NA	NA
AE	ACE	3	354.365	241	-127.635	0.976	1	0.323
CE	ACE	3	354.738	241	-127.262	1.349	1	0.245
E	ACE	2	355.125	242	-128.875	1.736	2	0.420

Supplemental Table 2c. Model fit statistics for *G. vaginalis* in the European American sample.

Model	Comparison	Parameters	-2*Log Likelihood	DF	AIC	Log Likelihood Difference	DF Difference	P-value
ACE	-	4	131.799	240	-348.201	NA	NA	NA
AE	ACE	3	132.421	241	-349.579	0.622	1	0.430
CE	ACE	3	131.889	241	-350.111	0.090	1	0.765
E	ACE	2	134.841	242	-349.159	3.042	2	0.219

Supplemental Table 2d. Model fit statistics for *L. crispatus* in the African American sample.

Model	Comparison	Parameters	-2*Log Likelihood	DF	AIC	Log Likelihood Difference	DF Difference	P-value
ACE	-	4	132.505	84	-35.495	NA	NA	NA
AE	ACE	3	132.608	85	-37.392	0.103	1	0.748
CE	ACE	3	132.854	85	-37.146	0.349	1	0.555
E	ACE	2	133.369	86	-38.631	0.864	2	0.649

Supplemental Table 2e. Model fit statistics for *L. iners* in the African American sample.

Model	Comparison	Parameters	-2*Log Likelihood	DF	AIC	Log Likelihood Difference	DF Difference	P-value
ACE	-	4	145.090	84	-22.910	NA	NA	NA
AE	ACE	3	145.103	85	-24.897	0.013	1	0.909
CE	ACE	3	145.141	85	-24.859	0.051	1	0.822
E	ACE	2	145.244	86	-26.756	0.155	2	0.926

Supplemental Table 2f. Model fit statistics for *G. vaginalis* in the African American sample.

Model	Comparison	Parameters	-2*Log Likelihood	DF	AIC	Log Likelihood Difference	DF Difference	P-value
ACE	-	4	38.507	84	-129.493	NA	NA	NA
AE	ACE	3	39.442	85	-130.558	0.935	1	0.334
CE	ACE	3	39.778	85	-130.222	1.271	1	0.260
E	ACE	2	39.894	86	-132.106	1.386	2	0.500

Supplemental Table 3a. Expanded list of taxa in European American MZ.

Taxa	ICC	95% LI	95% UI	*Cell 11	Cell 12	Cell 21	Cell 22
Aerococcus.christensenii	0.0401	-0.1405	0.2182	39	21	14	10
Anaerococcus.OTU145	0.0327	-0.1478	0.2111	48	9	21	6
Anaerococcus.tetradius	0.0000	-0.1796	0.1796	63	9	11	1
Atopobium.vaginae	0.3185	0.1473	0.4712	22	22	18	22
Bifidobacterium.breve_cluster	0.3262	0.1557	0.4778	57	15	8	4
Clostridiales.BVAB2	0.0000	-0.1796	0.1796	55	14	13	2
Coriobacteriaceae.OTU27	0.0000	-0.1796	0.1796	46	14	18	6
Dialister.cluster51	0.0000	-0.1796	0.1796	50	15	14	5
Enterobacteriaceae.cluster31	0.0232	-0.1570	0.2020	50	10	13	11
Finegoldia.magna	0.5713	0.4364	0.6810	21	20	18	25
Gardnerella.vaginalis	0.0000	-0.1796	0.1796	1	13	8	62
Lactobacillus.crispatus_cluster	0.3801	0.2152	0.5240	3	12	10	59
Lactobacillus.gasseri_cluster	0.1638	-0.0163	0.3336	22	17	20	25
Lactobacillus.iners	0.1245	-0.0564	0.2975	1	7	9	67
Lactobacillus.jensenii	0.0000	-0.1796	0.1796	22	25	13	24
Megasphaera.cluster52	0.0000	-0.1796	0.1796	45	17	12	10
Mycoplasma.hominis	0.0172	-0.1630	0.1962	54	14	9	7
Parvimonas.OTU142	0.0000	-0.1796	0.1796	55	12	13	4
Peptoniphilus.indolicus	0.2794	0.1051	0.4371	39	16	15	14
Prevotella.OTU48	0.1578	-0.0224	0.3281	66	4	11	3
Prevotella.amnii	0.0000	-0.1796	0.1796	51	16	12	5
Prevotella.bivia	0.0000	-0.1796	0.1796	29	18	22	15
Prevotella.cluster2	0.0000	-0.1796	0.1796	17	15	20	32
Prevotella.disiens	0.0938	-0.0872	0.2689	50	13	16	5
Sneathia.amnii	0.0439	-0.1368	0.2218	42	12	16	14
Sneathia.sanguinegens	0.0000	-0.1796	0.1796	58	12	8	6
Staphylococcus.cluster47	0.0104	-0.1696	0.1896	36	16	15	17
Streptococcus.agalactiae	0.0041	-0.1756	0.1836	56	9	15	4
Streptococcus.anginosus	0.1194	-0.0616	0.2927	42	14	17	11
TM7.OTU.H1	0.0000	-0.1796	0.1796	59	9	12	4
Ureaplasma.cluster23	0.1010	-0.0800	0.2757	39	11	15	19
X_Lachnospiraceae_.BVAB1	0.0000	-0.1796	0.1796	23	22	18	21

* Cell XX denotes presence/absence of taxa in 2x2 contingency table within twin pair
1 = Absent; 2 = Present; Cell 11 = taxa absent in both members of a twin pair
ICC = Intraclass Correlation; LI = Lower Interval; UI = Upper Interval

Supplemental Table 3b. Expanded list of taxa in European Amerrrican DZ twins.

Taxa	ICC	95% LI	95% UI	*Cell 11	Cell 12	Cell 21	Cell 22
Aerococcus.christensenii	0.0000	-0.2673	0.2673	15	10	8	5
Anaerococcus.OTU145	0.2025	-0.0684	0.4457	23	6	6	3
Anaerococcus.tetradius	0.0000	-0.2673	0.2673	27	8	3	0
Atopobium.vaginae	0.0000	-0.2673	0.2673	12	6	8	12
Bifidobacterium.breve_cluster	0.1026	-0.1693	0.3600	30	3	4	1
Clostridiales.BVAB2	0.0000	-0.2673	0.2673	28	5	3	2
Coriobacteriaceae.OTU27	0.0000	-0.2673	0.2673	22	9	6	1
Dialister.cluster51	0.0595	-0.2112	0.3216	19	10	6	3
Enterobacteriaceae.cluster31	0.0080	-0.2598	0.2747	26	5	6	1
Finegoldia.magna	0.4875	0.2532	0.6678	7	7	12	12
Gardnerella.vaginalis	0.0000	-0.2673	0.2673	2	6	4	26
Lactobacillus.crispatus_cluster	0.0000	-0.2673	0.2673	2	3	4	29
Lactobacillus.gasseri_cluster	0.0000	-0.2673	0.2673	10	7	11	10
Lactobacillus.iners	0.0000	-0.2673	0.2673	1	4	3	30
Lactobacillus.jensenii	0.0870	-0.1846	0.3462	8	8	8	14
Megasphaera.cluster52	0.1120	-0.1601	0.3682	20	8	5	5
Mycoplasma.hominis	0.0000	-0.2673	0.2673	23	6	8	1
Parvimonas.OTU142	0.0000	-0.2673	0.2673	22	8	6	2
Peptoniphilus.indolicus	0.3929	0.1403	0.5974	14	8	12	4
Prevotella.OTU48	0.0000	-0.2673	0.2673	27	6	4	1
Prevotella.amnii	0.0081	-0.2597	0.2748	28	4	3	3
Prevotella.bivia	0.0000	-0.2673	0.2673	14	10	11	3
Prevotella.cluster2	0.1689	-0.1030	0.4173	13	4	6	15
Prevotella.disiens	0.5215	0.2955	0.6923	29	4	3	2
Sneathia.amnii	0.0000	-0.2673	0.2673	16	5	10	7
Sneathia.sanguinegens	0.0752	-0.1960	0.3357	28	6	1	3
Staphylococcus.cluster47	0.4595	0.2192	0.6473	15	5	10	8
Streptococcus.agalactiae	0.0000	-0.2673	0.2673	25	8	4	1
Streptococcus.anginosus	0.1691	-0.1028	0.4175	18	8	7	5
TM7.OTU.H1	0.0000	-0.2672	0.2673	26	6	4	2
Ureaplasma.cluster23	0.0024	-0.2650	0.2695	16	10	5	7
X_Lachnospiraceae_.BVAB1	0.0495	-0.2207	0.3126	14	12	5	7

* Cell XX denotes presence/absence of taxa in 2x2 contingency table within twin pair
1 = Absent; 2 = Present; Cell 11 = taxa absent in both members of a twin pair
ICC = Intraclass Correlation; LI = Lower Interval; UI = Upper Interval

Supplemental Table 3c. Expanded list of taxa in African American MZ.

Taxa	ICC	95% LI	95% UI	*Cell 11	Cell 12	Cell 21	Cell 22
Aerococcus.christensenii	0.5498	0.2616	0.7479	5	3	7	9
Anaerococcus.OTU145	0.0179	-0.3205	0.3523	13	5	3	3
Anaerococcus.tetradius	0.1355	-0.2106	0.4515	13	5	2	4
Atopobium.vaginae	0.0936	-0.2508	0.4170	2	7	6	9
Bifidobacterium.breve_cluster	0.0000	-0.3365	0.3365	13	3	7	1
Clostridiales.BVAB2	0.0000	-0.3365	0.3365	8	5	8	3
Coriobacteriaceae.OTU27	0.2473	-0.0973	0.5390	10	4	2	8
Dialister.cluster51	0.0000	-0.3365	0.3365	5	6	7	6
Enterobacteriaceae.cluster31	0.0000	-0.3365	0.3365	18	2	3	1
Finegoldia.magna	0.1251	-0.2207	0.4430	11	1	4	8
Gardnerella.vaginalis	0.2250	-0.1207	0.5220	0	2	0	22
Lactobacillus.crispatus_cluster	0.2045	-0.1417	0.5062	3	1	7	13
Lactobacillus.gasseri_cluster	0.0000	-0.3365	0.3365	6	8	8	2
Lactobacillus.iners	0.0946	-0.2499	0.4178	0	1	1	22
Lactobacillus.jensenii	0.6247	0.3649	0.7943	6	6	10	2
Megasphaera.cluster52	0.0000	-0.3365	0.3365	6	5	7	6
Mycoplasma.hominis	0.0578	-0.2842	0.3868	10	5	6	3
Parvimonas.OTU142	0.0580	-0.2841	0.3870	10	5	5	4
Peptoniphilus.indolicus	0.1152	-0.2303	0.4348	7	7	3	7
Prevotella.OTU48	0.1318	-0.2143	0.4484	17	1	4	2
Prevotella.amnii	0.0000	-0.3365	0.3365	9	5	5	5
Prevotella.bivia	0.1193	-0.2263	0.4382	4	6	9	5
Prevotella.cluster2	0.0737	-0.2695	0.4003	4	3	5	12
Prevotella.disiens	0.0467	-0.2944	0.3773	14	3	3	4
Sneathia.amnii	0.1222	-0.2235	0.4406	7	5	5	7
Sneathia.sanguinegens	0.0000	-0.3365	0.3365	9	3	6	6
Staphylococcus.cluster47	0.0000	-0.3365	0.3365	7	2	11	4
Streptococcus.agalactiae	0.0059	-0.3313	0.3417	11	3	7	3
Streptococcus.anginosus	0.1423	-0.2040	0.4569	10	4	7	3
TM7.OTU.H1	0.0000	-0.3365	0.3365	14	3	6	1
Ureaplasma.cluster23	0.0000	-0.3365	0.3365	3	4	6	11
X_Lachnospiraceae_.BVAB1	0.0000	-0.3365	0.3365	8	3	5	8

* Cell XX denotes presence/absence of taxa in 2x2 contingency table within twin pair
1 = Absent; 2 = Present; Cell 11 = taxa absent in both members of a twin pair
ICC = Intraclass Correlation; LI = Lower Interval; UI = Upper Interval

Supplemental Table 3d. Expanded list of taxa in African American DZ twins.

Taxa	ICC	95% LI	95% UI	*Cell 11	Cell 12	Cell 21	Cell 22
Aerococcus.christensenii	0.0000	-0.3687	0.3687	5	4	6	5
Anaerococcus.OTU145	0.0000	-0.3687	0.3687	13	3	2	2
Anaerococcus.tetradius	0.0000	-0.3687	0.3687	9	3	6	2
Atopobium.vaginae	0.5096	0.1735	0.7394	2	4	4	10
Bifidobacterium.breve_cluster	0.0000	-0.3687	0.3687	16	2	1	1
Clostridiales.BVAB2	0.0000	-0.3687	0.3687	8	5	4	3
Coriobacteriaceae.OTU27	0.0000	-0.3687	0.3687	7	7	3	3
Dialister.cluster51	0.0000	-0.3687	0.3687	4	7	6	3
Enterobacteriaceae.cluster31	0.0935	-0.2851	0.4468	13	2	3	2
Finegoldia.magna	0.0000	-0.3687	0.3687	6	6	5	3
Gardnerella.vaginalis	0.0000	-0.3687	0.3687	0	3	2	15
Lactobacillus.crispatus_cluster	0.0622	-0.3137	0.4213	3	2	5	10
Lactobacillus.gasseri_cluster	0.0000	-0.3687	0.3687	6	5	2	7
Lactobacillus.iners	0.0304	-0.3422	0.3947	0	0	0	20
Lactobacillus.jensenii	0.0000	-0.3687	0.3687	7	1	5	7
Megasphaera.cluster52	0.0000	-0.3687	0.3687	5	7	4	4
Mycoplasma.hominis	0.0306	-0.3420	0.3949	12	4	2	2
Parvimonas.OTU142	0.0000	-0.3687	0.3687	8	6	4	2
Peptoniphilus.indolicus	0.0243	-0.3476	0.3895	8	6	5	1
Prevotella.OTU48	0.0000	-0.3687	0.3687	12	3	4	1
Prevotella.amnii	0.0000	-0.3687	0.3687	11	4	4	1
Prevotella.bivia	0.1428	-0.2385	0.4860	5	4	6	5
Prevotella.cluster2	0.0000	-0.3687	0.3687	4	5	3	8
Prevotella.disiens	0.0000	-0.3687	0.3687	14	3	2	1
Sneathia.amnii	0.0000	-0.3687	0.3687	6	9	2	3
Sneathia.sanguinegens	0.0000	-0.3687	0.3687	6	6	5	3
Staphylococcus.cluster47	0.0000	-0.3687	0.3687	8	5	3	4
Streptococcus.agalactiae	0.0288	-0.3436	0.3934	13	2	3	2
Streptococcus.anginosus	0.0000	-0.3687	0.3687	12	5	3	0
TM7.OTU.H1	0.0000	-0.3687	0.3687	13	4	2	1
Ureaplasma.cluster23	0.0000	-0.3687	0.3688	8	1	5	6
X_Lachnospiraceae_.BVAB1	0.1208	-0.2595	0.4686	7	5	1	7

* Cell XX denotes presence/absence of taxa in 2x2 contingency table within twin pair
1 = Absent; 2 = Present; Cell 11 = taxa absent in both members of a twin pair
ICC = Intraclass Correlation; LI = Lower Interval; UI = Upper Interval
