# Supplementary material for: Vaginal microbiome Lactobacillus crispatus is heritable among European American women
Source: Commun Biol. 2021 Aug 5;4:872. doi: 10.1038/s42003-021-02394-6 (PMC8342574; doi:10.1038/s42003-021-02394-6)
Supplement: Supplementary file 3 — Reporting Summary [file 42003_2021_2394_MOESM3_ESM.pdf]

nature research

Corresponding author(s): Timothy P. York

Last updated by author(s): Oct 27, 2020

Reporting Summary

Nature Research wishes to improve the reproducibility of the work that we publish. This form provides structure for consistency and transparency in reporting. For further information on Nature Research policies, see our Editorial Policies and the Editorial Policy Checklist.

Statistics

For all statistical analyses, confirm that the following items are present in the figure legend, table legend, main text, or Methods section.

n/a	Confirmed	
☐	☒	The exact sample size (n) for each experimental group/condition, given as a discrete number and unit of measurement
☐	☒	A statement on whether measurements were taken from distinct samples or whether the same sample was measured repeatedly
☐	☒	The statistical test(s) used AND whether they are one- or two-sided<br>*Only common tests should be described solely by name; describe more complex techniques in the Methods section.*
☐	☒	A description of all covariates tested
☐	☒	A description of any assumptions or corrections, such as tests of normality and adjustment for multiple comparisons
☐	☒	A full description of the statistical parameters including central tendency (e.g. means) or other basic estimates (e.g. regression coefficient) AND variation (e.g. standard deviation) or associated estimates of uncertainty (e.g. confidence intervals)
☐	☒	For null hypothesis testing, the test statistic (e.g. F, t, r) with confidence intervals, effect sizes, degrees of freedom and P value noted<br>*Give P values as exact values whenever suitable.*
☒	☐	For Bayesian analysis, information on the choice of priors and Markov chain Monte Carlo settings
☐	☒	For hierarchical and complex designs, identification of the appropriate level for tests and full reporting of outcomes
☒	☐	Estimates of effect sizes (e.g. Cohen's d, Pearson's r), indicating how they were calculated

Our web collection on statistics for biologists contains articles on many of the points above.

Software and code

Policy information about availability of computer code

Data collection	Data used for this study has been uploaded to dbGAP under Study Accession number phs000256.
Data analysis	Code to compute heritability can be accessed at https://github.com/tpyork/twin-microbiome.

For manuscripts utilizing custom algorithms or software that are central to the research but not yet described in published literature, software must be made available to editors and reviewers. We strongly encourage code deposition in a community repository (e.g. GitHub). See the Nature Research guidelines for submitting code & software for further information.

Data

Policy information about availability of data

All manuscripts must include a data availability statement. This statement should provide the following information, where applicable:

- Accession codes, unique identifiers, or web links for publicly available datasets
- A list of figures that have associated raw data
- A description of any restrictions on data availability

Data used for this study has been uploaded to dbGAP under Study Accession number phs000256.

Field-specific reporting

Behavioural & social sciences study design

All studies must disclose on these points even when the disclosure is negative.

Study description	Quantitative cross-sectional
Research sample	Adult female twin pairs over age 18 were recruited from the Mid-Atlantic Twin Registry in Richmond Virginia. Women included in this study were of self-reported European or African Ancestry. Data was collected during a previous study and details are available here: https://www.nature.com/articles/npre.2010.5150.1 & https://pubmed.ncbi.nlm.nih.gov/25073854/
Sampling strategy	Twin pairs were randomly ascertained within race and zygosity class from the Mid-Atlantic Twin Registry. Sample size was estimated by the excess of the intra-pair variance of DZ twins over that within MZ twins that can be quantified from the F-test (Vandenberg, 1966). Under the assumption that genetic effects are additive, F is a function of the heritability of the trait (in our case prevalence of the species in individual twins). The model used for the power calculations assumes that gene effects are additive, mating is random and that environmental effects are not correlated between twins (Jinks and Fulker, 1970). It was assumed that the raw data were continuous and can be transformed to normality. Power would be lower for presence/absence data. It was determined that samples of 100-200 pairs (similar to our reported sample size) would be required to detect moderate genetic effects on individual species.
Data collection	Participants filled out a detailed questionnaire that included questions about ethnicity, education, employment, health habits, dietary habits and sexual history. Clinicians also filled out a diagnosis form at the time of each visit that included information about the purpose of each visit, and any diagnoses. Subjects were considered 'healthy' at the time of a visit if the purpose of the visit was for an annual examination, they received no diagnosis and were asymptomatic (e.g. no abnormal discharge). BV testing was performed only when indicated, and was based solely on Amsel's criteria (Amsel et al., 1983). Samples were taken by a physician using CultureSwab EZ (Becton Dickinson) from the mid-vaginal wall during a speculum examination. DNA was extracted from the swabs within 4 h of collection using the Powersoil kit (MoBio). The swabs were swirled directly in the Powerbead tubes supplied with the kit and processing was performed according to the manufacturer's instructions.
Timing	2009–2013
Data exclusions	A total of 380 mid-vaginal wall swabs were obtained from self-identified MZ or DZ twin participants (Table 1). Thirty-four samples were collected from only one member of a twin pair and not included in these analyses. 7 twin pairs did not identify as African or European ancestry and were not included.
Non-participation	This is a sub-set of data from the original study mentioned above. Only twins with zygosity and ancestry included in the original study were included for these analysis.
Randomization	Not applicable. Participants were allocated to groups based on self-identified ancestry.

Reporting for specific materials, systems and methods

We require information from authors about some types of materials, experimental systems and methods used in many studies. Here, indicate whether each material, system or method listed is relevant to your study. If you are not sure if a list item applies to your research, read the appropriate section before selecting a response.

Materials & experimental systems

n/a	Involved in the study	
☒	☐	Antibodies
☒	☐	Eukaryotic cell lines
☒	☐	Palaeontology and archaeology
☒	☐	Animals and other organisms
☐	☒	Human research participants
☒	☐	Clinical data
☒	☐	Dual use research of concern

Methods

n/a	Involved in the study	
☒	☐	ChIP-seq
☒	☐	Flow cytometry
☒	☐	MRI-based neuroimaging

Human research participants

Policy information about studies involving human research participants

Population characteristics	There were 322 twins of self-identified African (44 pairs) or European ancestry (122 pairs). There was a higher proportion of African ancestry DZ than MZ twins (chi-square = 5.21, P-value = 0.022). Participants ranged in age from 18 to 78 years old (median = 36). The median BMI of all participants was 27 (range = 17 - 50), and the mean was higher among participants of African ancestry (t-test = 4.45, P-value < 0.001). In this sample participants of African ancestry were more often diagnosed with bacterial vaginosis (chi-square = 9.48, P-value = 0.002), while more participants of European ancestry reported active smoking at the time of assessment (chi-square = 6.66, P-value = 0.010).
Recruitment	Participants were recruited in 2009–2013 from outpatient clinics at the Virginia Commonwealth University (VCU) Medical Center and the Virginia Department of Health following written, informed consent. Inclusion criteria included women age 18–44 years who were able to provide informed consent and who were willing or already scheduled to undergo a vaginal examination using a speculum. Participants filled out a detailed questionnaire that included questions about ethnicity, education, employment, health habits, dietary habits and sexual history. Participants who self-reported African American (black) race and not Latino ethnicity are referred to as African Ancestry. Women who self-reported race as Caucasian (white) and not Latino ethnicity are referred to as women of European ancestry. Clinicians also filled out a diagnosis form at the time of each visit that included information about the purpose of each visit, and any diagnoses. Subjects were considered 'healthy' at the time of a visit if the purpose of the visit was for an annual examination, they received no diagnosis and were asymptomatic (e.g. no abnormal discharge). BV testing was performed only when indicated, and was based solely on Amsel's criteria (Amsel et al., 1983).
Ethics oversight	The Institutional Review Boards for Human Subjects Research at VCU (Panel B) and the Virginia Department of Health reviewed and approved this study.

Note that full information on the approval of the study protocol must also be provided in the manuscript.
